# Palmelloid formation in the Antarctic psychrophile, *Chlamydomonas priscuii*, is photoprotective

**DOI:** 10.3389/fpls.2022.911035

**Published:** 2022-08-31

**Authors:** Beth Szyszka-Mroz, Alexander G. Ivanov, Charles G. Trick, Norman P. A. Hüner

**Affiliations:** ^1^Department of Biology and the Biotron Centre for Experimental Climate Change Research, University of Western Ontario, London, ON, Canada; ^2^Institute of Biophysics and Biomedical Engineering, Bulgarian Academy of Sciences, Sofia, Bulgaria; ^3^School of Public Health, University of Saskatchewan, Saskatoon, SK, Canada

**Keywords:** *Chlamydomonas priscuii*, antarctic, psychrophile, palmelloids, photoprotection, temperature

## Abstract

Cultures of the obligate, Antarctic psychrophile, *Chlamydomonas priscuii* grown at permissive low temperature (8°C) are composed of flagellated, single cells, as well as non-motile, multicellular palmelloids. The relative proportions of the two cell types are temperature dependent. However, the temperature dependence for palmelloid formation is not restricted to psychrophilic *C. priscuii* but appears to be a general response of mesophilic Chlamydomonas species (*C. reinhardtii* and *C. raudensis*) to non-permissive growth temperatures. To examine potential differences in photosynthetic performance between single cells versus palmelloids of the psychrophile, a cell filtration technique was developed to separate single cells from palmelloids of *C. priscuii* grown at 8°C. Flow cytometry was used to estimate the diameter of isolated single cells (≤5 μm) versus isolated palmelloids of varying size (≥8 μm). Compared to single cells, palmelloids of *C. priscuii* showed a decrease in the abundance of light-harvesting complex II (LHCII) proteins with a 2-fold higher Chl a/b ratio. A decrease in both lutein and β-carotene in palmelloids resulted in carotenoid pools which were 27% lower in palmelloids compared to single cells of the psychrophile. Chlorophyll fluorescence analyses of the isolated fractions revealed that maximum photochemical efficiency of PSII (F_v_/F_m_) was comparable for both single cells and palmelloids of *C. priscuii*. However, isolated palmelloids exhibited lower excitation pressure, measured as 1 - qL, but higher yield of PSII (Φ_PSII_) and 50% higher rates of electron transport (ETR) than single cells exposed to high light at 8°C. This decreased sensitivity to high light in isolated palmelloids compared to single cells was associated with greater non-regulated dissipation of excess absorbed energy (Φ_NO_) with minimal differences in Φ_NPQ_ in *C. priscuii* in response to increasing irradiance at low temperature. The ratio Φ_NO_/Φ_NPQ_ observed for isolated palmelloids of *C. priscuii* developed at 8°C (1.414 ± 0.036) was 1.38-fold higher than Φ_NO_/Φ_NPQ_ of isolated single cells (1.021 ± 0.018) exposed to low temperature combined with high light (1,000 μmol m^−2^ s^−1^). The differences in the energy quenching capacities between palmelloids and single cells are discussed in terms of enhanced photoprotection of *C. priscuii* palmelloids against low-temperature photoinhibition.

## Introduction

*Chlamydomonas reinhardtii* is the model unicellular, biflagellate green alga commonly used in the study of algal photosynthesis (Rochaix, 1995, 2013, 2014; Eberhard et al., 2008; Harris, 2009). During asexual reproduction, *C. reinhardtii* protoplasts can divide into 2, 4, 8, or 16 daughter cells from a single mother cell. Prior to the division, cells can double several times in size, and the number of cell divisions depends on the size of the mother cell, resulting in the formation of unicellular daughter cells that are uniform in size (Craigie and Cavalier-Smith, 1982; Oldenhof et al., 2007). During sexual reproduction, fusion of gametes leads to the formation of a zygospore, a specialized cell surrounded by a hard, thick, multilayered wall, which is resistant to harsh environmental conditions (Trainor, 1985; Harris, 2009). Among some species of *Chlamydomonas*, akinete (asexual resting spore) formation has been reported under unfavorable conditions such as desiccation stress where the vegetative cell wall thickens, concomitant with the accumulation of carotenoids, starch, and lipids (Coleman, 1983).

A third morphological variant has also been observed. “Palmelloid cells” is a broad term that describes a morphological state characterized by multiple, single cells encased in an outer limiting membrane (Pocock et al., 2004). The presence of “palmelloid cells” has been observed among species of *Chlamydomonas* to varying degrees (Harris, 2009). Previous reports indicate that palmelloid formation in *Chlamydomonas* can be induced by various conditions including the presence of chelating agents, non-metabolizable organic acids, calcium deficiency, high phosphate concentrations, and exposure to low pH values in the growth medium (Iwasa and Murakami, 1968, 1969; Visviki and Santikul, 2000; Harris, 2009). Olsen et al. (1983) observed that during growth under phosphate limitation, palmelloids of *C. reinhardtii* exhibited lower growth rates (0.03 h^−1^) compared to free-swimming, vegetative cells (0.21 h^−1^). Exposure to the herbicide paraquat which inhibits photosynthetic electron transport at photosystem I (Devine et al., 1993) also induced palmelloid formation in *C. eugametos* (Franqueira et al., 1999). Paraquat results in oxidative stress due to the generation of superoxide anion, a reactive oxygen species (ROS), which causes lipid peroxidation and damage to cell membranes (Tissut et al., 1987). It is presumed that palmelloids result from a failure of the daughter cells to be released from the mother cell, due to either flagellar malfunction or abnormal production of membranes and cell walls (Fritsch, 1945; Nakamura et al., 1975; Harris, 2009). In *C. eugametos*, the palmelloid condition can be induced by chloroplatinic acid, a compound that blocks cell division and causes incomplete cytokinesis (Rosenberg et al., 1967; Nakamura et al., 1975). Palmelloids produced in this way showed multiple layers of cell wall material, and it was concluded that the inability of palmelloid cells to separate in this case, occurs due to abnormalities in cell wall formation (Nakamura et al., 1975).

In synchronous cultures grown under a 12 h light-12 h dark regime, the *Chlamydomonas reinhardtii* mutant ‘ls’, exhibited delayed, light-dependent liberation of zoospores (Mergenhagen, 1980). It was suggested that this light dependency was due to a deficiency in energy supply in the dark since it could be overcome by an extended light period or the addition of acetate (Voigt et al., 1989). Furthermore, this mutant accumulates sporangia under suboptimal aeration and in high-density cultures entering the stationary phase (Voigt et al., 1989). In this case, increasing the light period or addition of a carbon source did not result in the release of zoospores, which occurred only after sporangia were resuspended in fresh culture medium (Voigt et al., 1989). Analyses of these sporangia revealed that they contained abnormal, multilayered sporangial walls, which were not observed under optimal growth conditions (Voigt et al., 1990). Studies have also suggested that the increased incidence of palmelloids in *Chlamydomonas* is due to the presence of grazers. The larger cells may provide protection from predation by rotifers by minimizing the risk of being consumed due to their size (Mikheeva and Kruchkova, 1980; Lurling and Beekman, 2006). Lurling and Beekman (2006) found that despite grazer presence, palmelloids were absent in the dark, suggesting that light and active growth are requirements for palmelloid formation. Furthermore, it has been suggested that formation of multicellular structures such as palmelloids is one strategy used by *Chlamydomonas* to acclimate and survive various environmental stresses (de Carpentier et al., 2019), including acclimation to low temperatures (Ermilova, 2020). de Carpentier et al. (2019) suggest that there is a link between stress responses, evolution, and the transition from a unicellular to a multicellular, colonial structure in *Chlamydomonas*. However, we have minimal understanding of the molecular mechanism(s) that govern palmelloid formation and the functional role of palmelloids as a phenotypic response to environmental stress.

*Chlamydomonas priscuii* was isolated from the permanently ice-covered Lake Bonney in Antarctica (77.7333° S, 162.1667° E) at a depth of 17 m. The thick, 3–4.5 m layer of ice cover attenuates between 97 and 99% incident PAR, absorbs wavelengths greater than 600 nm and prevents vertical mixing of the water column (Howard-Williams et al., 1998; Fritsen and Priscu, 1999). Consequently, Lake Bonney consists of highly stratified layers of phytoplankton (Spigel and Priscu, 1996). The discrete position of *C. priscuii* in the water column is characterized by low irradiance (<15 μmol photons m^−2^ s^−1^) in the blue–green range, high salinity, and average annual temperatures between 4 and 6°C (Lizotte and Priscu, 1994). *C. priscuii* is classified as an obligate psychrophile with an optimal growth temperature of 8–12°C but an inability to grow above 20°C (Cvetkovska et al., 2022). Despite the extremely stable environment of Lake Bonney, this shade-adapted psychrophile exhibits the capacity to acclimate to alterations in temperature and light regimes (Morgan et al., 1998; Morgan-Kiss et al., 2002; Szyszka et al., 2007). Cells of *C. priscuii* are biflagellate, oval-shaped, and exist as either free-swimming, motile single cells or as non-motile, membrane-bound, palmelloids that encase multiple, flagellated single cells (Pocock et al., 2004).

All photosynthetic research on the Antarctic psychrophile, *C. priscuii*, has been performed on heterogeneous populations containing both motile, single cells as well as colonial palmelloids. It has been assumed that the single chloroplast within a motile, single cell of *C. priscuii* is functionally and compositionally equivalent to a chloroplast localized in the single cells encased within a palmelloid. To address this assumption, we developed a simple method to isolate single cells from palmelloids obtained from the same culture. By a comparison of the photosynthetic characteristics of physically separated single cells from palmelloids of *C. priscuii* grown at permissive low temperature (8°C), we show that palmelloids are photosynthetically distinct from single cells and that palmelloid formation is photoprotective for this psychrophile.

## Materials and methods

### Growth conditions

Mesophilic *Chlamydomonas reinhardtii* (1690) and *Chlamydomonas raudensis* SAG 49.7 were grown axenically in Bold’s basal medium (BBM), whereas the obligate psychrophile, *Chlamydomonas priscuii,* was grown in BBM supplemented with 0.7 M NaCl. All cultures were aerated continuously under ambient CO_2_ conditions in 250 ml glass Pyrex tubes suspended in temperature-regulated aquaria. Growth irradiance was generated by fluorescent tubes (Sylvania CW-40) and measured with a quantum sensor attached to a radiometer (Model LI-189; Li-Cor, Lincoln, Neb., United States). Control *Chlamydomonas priscuii* cells were grown at 5°C, whereas growth temperature experiments were performed between 8°C and 16°C for *C. priscuii* and between 11 and 28°C for both *C. reinhardtii* 1690 and *C. raudensis* SAG 49.72. All cells were grown at an irradiance of 150 μmol photons m^−2^ s^−1^. Mid-log phase cells were used in all experiments.

### Cell filtration

Using a glass filter holder with fritted glass support (EMD Millipore), approximately 1 l of dilute cell culture was passed through 11 μm hydrophilic nylon net filters (EMD Millipore, product #NY1102500) in order to remove any large clumps of cells. During this first step, culture dilution was adjusted so that the cell suspension was able to pass through the apparatus by gravity flow alone. Cells captured on the filter were discarded and the flow-through filtrate was utilized as the control fraction. This control cell suspension was passed through hydrophilic polycarbonate membrane filters with 8 μm pores (Sterlitech, product #PCT8025100) by drawing fluid with the aid of gentle vacuum aspiration. Cells retained on the filters were washed three times with 4 ml of culture medium to resuspend the cells. Resulting filters were collected in Falcon tubes and suspended with 5 ml of media. The supernatant was centrifuged at 3000 rpm for 5 min to concentrate the cells which were used as the 8–11 μm fraction. The flow-through filtrate was passed through hydrophilic polycarbonate membrane filters with 5 μm pores (Sterlitech, product #PCT5025100) using gentle vacuum aspiration. Cells retained on the filters were washed, resuspended in culture medium and centrifuged as above, and represented the 5–8 μm fraction, whereas, the flow-through filtrate was centrifuged and represented the single cell, <5 μm fraction.

### Flow cytometry

Control cells and separated cell fractions were diluted and analyzed using a BD Accuri^TM^ C6 flow cytometer (BD Biosciences, Oxford, United Kingdom). Cell sizes were examined through forward scatter (FSC) and red chlorophyll fluorescence (FL3). Analysis of the mean FSC and FL3 values was performed by manual gating to exclude debris at lower scatter intensities. Culture samples were analyzed in triplicate, using a volume of 50 μl, with a minimum of 10,000 events collected for each sample. Results were visualized by the creation of FSC and FL3 overlay histograms.

### Low-temperature (77 K) chlorophyll fluorescence spectroscopy

Low-temperature (77 K) chlorophyll fluorescence emission spectra of *C. priscuii* cell cultures and cell fractions were collected as described previously (Szyszka et al., 2007) using a PTI QM-7/2006 spectrofluorometer (Photon Technology International, South Brunswick, NJ, United States) equipped with double monochromators, R928P red-sensitive photomultiplier tube (Hamamatsu Photonics, Shizuoka-ken, Japan) and a liquid nitrogen cuvette. Cell cultures and cell fractions were frozen in liquid nitrogen in the presence of 30% (v/v) glycerol before the measurements. Corrected fluorescence emission spectra were excited at 436 nm and recorded from 650 nm to 800 nm using slit width of 2.5 nm for both excitation and emission. The fluorescence difference spectra were obtained as described in (Savitch et al., 2011).

Decomposition analysis of the fluorescence emission spectra in terms of 5 Gaussian bands was carried out by a non-linear least squares algorithm that minimizes the chi-square function using a Microcal Origin Version 6.0 software package (Microcal Software, Northampton, MA, United States). The fitting parameters for the five Gaussian components, that is, position, area, and full width at the half-maximum (FWHM), were free-running parameters.

### Room temperature chlorophyll fluorescence induction

*In vivo*, room temperature modulated Chl *a* fluorescence was measured in control cultures, isolated single cells, and isolated palmelloids (10 μg Chl mL^−1^) using a Xe-PAM system (Heinz-Walz GmbH, Effeltrich, Germany). After 15 min of dark adaptation, Chl fluorescence at open PSII reaction centers (F_o_) was excited by a non-actinic modulated measuring beam (0.5 μmol m^−2^ s^−1^), pulsed at 2 Hz. Maximum fluorescence at closed PSII reaction centers (F_m_) was induced by a saturating white light pulse (800 ms, 2,400 μmol photons m^−2^ s^−1^). All measurements were performed at the corresponding growth temperature using a water-jacketed cuvette. The time frame for each light intensity was 6 min with a frequency of saturating flashes of 30s. The same cells were used for each measurement and allowed to relax for 2 min in the dark between the different light intensities. Maximum photochemical efficiency (F_v_/F_m_) was calculated as F_m_–F_o_/F_m_, using dark-adapted cells. F_m′_ was measured under constant actinic light (100 μmol m^−2^ s^−1^), with saturating light flashes in 30 s intervals. Data acquisition was managed using a PAM-Data Acquisition System PDA-100 and the WinControl software application (Heinz-Walz).

Fluorescence parameters were calculated during steady-state photosynthesis using the equations described by Kramer et al. (2004): Φ_PSII_ = (F_m_′–F_s_)/F_m_′, Φ_NO_ = 1/[NPQ + 1 + qL(F_m_/F_o_–1)], Φ_NPQ_ = 1–Φ_PSII_–Φ_NO_, where Φ_PSII_ is the yield of PSII photochemistry, Φ_NPQ_ is the yield of non-photochemical energy dissipation by down-regulation through antenna quenching and Φ_NO_ is the yield of all other processes involved in non-photochemical energy losses. The relative PSII electron transport rate was determined as ETR = Φ_PSII_ × (PFD × 0.84) × 0.5.

### Pigment analysis and epoxidation states

Algal cells were harvested by centrifugation and pigments were extracted by disrupting cells with 100% acetone at 4°C using a Mini-BeadBeater (3110BX, BioSpec). Extracts were clarified by centrifugation. The supernatant was filtered through a 0.22 μm syringe filter and samples were stored at −20°C until analyzed. Pigments were separated and quantified by high-performance liquid chromatography (HPLC) as described previously (Ivanov et al., 1995). The system contained a Beckman System Gold programmable solvent module 126, diode array detector module 168 (Beckman Instruments, San Ramon, CA, United States), CSC-Spherisorb ODS-1 reverse phase column (5 μm particle size, 25 × 0.46 cm I.D.) with an Upchurch Perisorb A guard column (both columns from Chromatographic Specialties Inc., Concord, ON, Canada). Samples were injected using a Beckman 210A sample injection valve with a 20 μl sample loop. Pigments were eluted isocratically for 6 min with a solvent system of acetonitrile:methanol: 0.1 M Tris–HCl (pH 8.0; 72:8:3.5, v/v/v), followed by a 2 min linear gradient to 100% methanol:hexane (75:25, v/v) which continued isocratically for 4 min. The total run time was 12 min. The flow rate was 2 cm^3^ min^−1^. Absorbance was detected at 440 nm, and peak areas were integrated by Beckman System Gold software. Retention times and response factors of Chl *a*, Chl *b*, lutein, and β-carotene were determined by injection of known amounts of pure standards purchased from Sigma (St. Louis, MO, United States). The retention times of zeaxanthin, antheraxanthin, and violaxanthin were determined by using pigments purified by thin-layer chromatography (Ivanov et al., 1995).

### SDS-PAGE and immunoblotting

Thylakoids were isolated as described in detail by Morgan et al. (1998). Thylakoid preparations were solubilized in a 60 mM Tris (pH 7.8) buffer containing 1 mm EDTA, 12% (w/v) sucrose, and 2% (w/v) SDS to attain an SDS:Chl ratio of 20:1. Samples were loaded on an equal Chl basis. Electrophoretic separation was performed with a 12.5% (w/v) polyacrylamide resolving gel containing 6 M urea, 0.66 M Tris (pH 8.8) and an 8% (w/v) polyacrylamide stacking gel containing 0.125 M Tris (pH 6.8) using the Laemmli buffer system (Laemmli, 1970). Electrophoresis was performed using a Mini-Protean II apparatus (Bio-Rad).

Separated thylakoid polypeptides were either stained with Coomassie Blue R, or transferred electrophoretically to nitrocellulose membranes (Bio-Rad, 0.2 μm pore size) at 5°C for 1 h at 100 V. The membranes were pre-blocked with a Tris-buffered salt (20 mm Tris, pH 7.5; 150 mm NaCl) containing 5% (w/v) milk powder and 0.01% (w/v) Tween 20. Membranes were probed with antibodies from Agrisera (AB, Vanas, Sweden). Lhcb4 (#AS06 117), Lhcbm5 (#AS09 408), Cyt f (#AS06 119), PsaA (#AS06 172), Lhcsr1 (#AS14-2819) and Lhcrs3 (#AS14-2766), antibodies were used, at a dilution of 1:10000, 1:5000, 1:5000, 1:2000, 1:1000, and 1:1000, respectively. After incubation with the secondary antibody conjugated with horseradish peroxidase (Sigma, 1:20000 dilution), the antibody-protein complexes were visualized by incubation in ECL chemiluminescent detection reagents (GE Healthcare) and developed on *X*-ray film (Fuji Film). Densitometric scanning and analysis of each replicate immunoblot was performed with a Hewlett Packard ScanJet 4200C desktop scanner and ImageJ 1.41o densitometry software (Wayne Rasband, National Institutes of Health, United States)^1^ as described earlier (Ivanov et al., 2015). Mean values ± SE were calculated from 3 independent experiments. The presented data were normalized to the relative abundance of PSI-and PSII-related proteins in WT plants.

## Results

### Growth temperature and cell morphology

**Figure 1:**
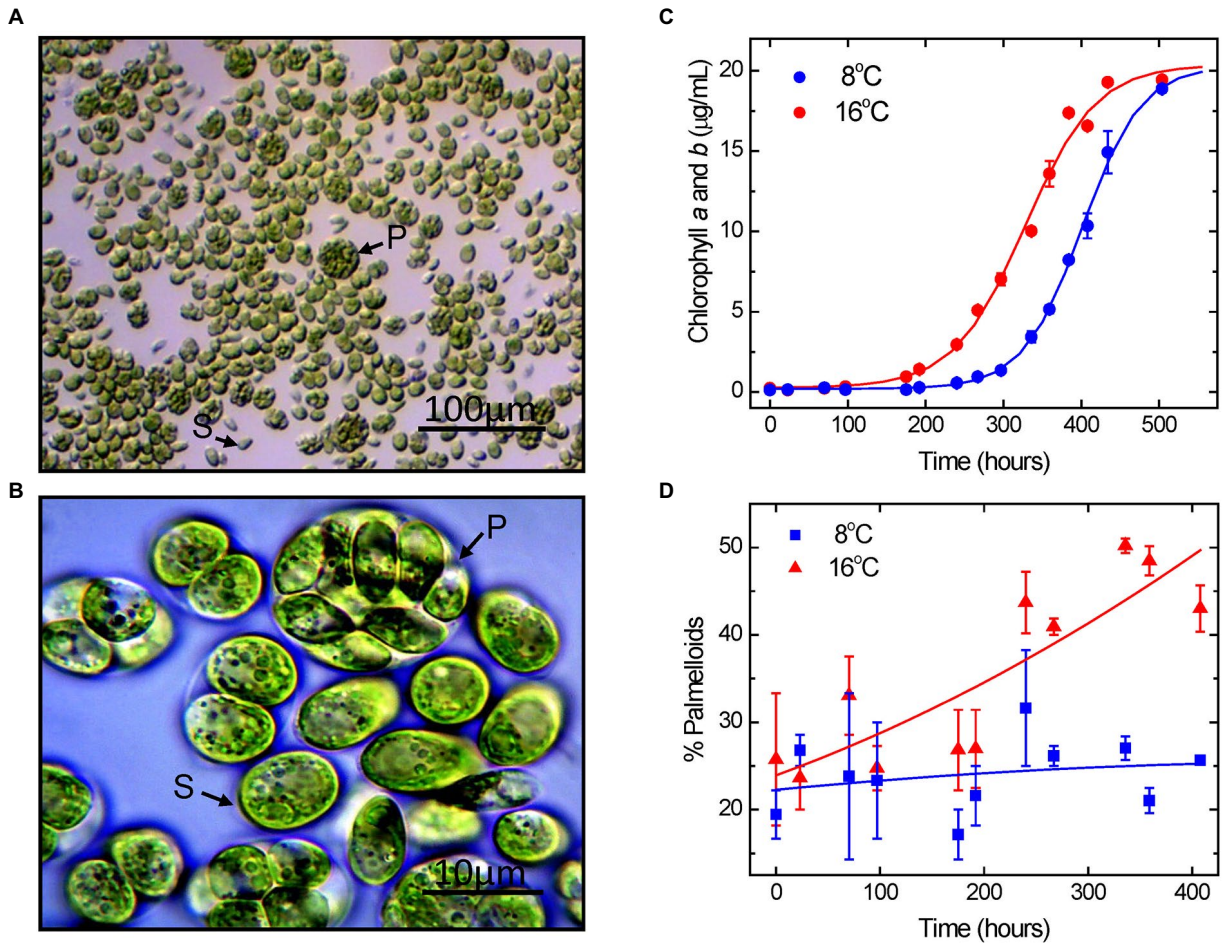
Light microscope images of *Chlamydomonas priscuii* cultures at 100x **(A)** and 1,000x **(B)** magnification. Cells exist as motile, flagellated single cells (S) and multicellular palmelloids (P). Growth curves of *C. priscuii* cells grown under steady-state temperatures of 8°C and 16°C, as measured by total chlorophyll concentrations **(C)**. *C. priscuii* exhibited an exponential growth rate of 0.0986 μg Chl h^−1^ at 8°C and 0.0483 μg Chl h^−1^ at 16°C. Percentage of cell units present as palmelloids was measured under the same growth temperature regimes **(D)**.

Figures 1A,B illustrate that growth of *Chlamydomonas priscuii* at low temperature (8°C) results in a heterogeneous population of single cells (Figures 1A,B, “S”) and palmelloids (Figures 1A,B, “P”). To assess whether growth temperature affects the proportion of the two cell types, *C. priscuii* was grown under steady-state temperatures either near their optimal low temperature of 8°C or at a supra-optimal temperature of 16°C at a common light intensity of light intensity of 150 μmol photons m^−2^ s^−1^ (Figure 1C). The growth rate of *C. priscuii* at 8°C (0.0986 μg Chl h^−1^) was about 2-fold higher than that observed at 16°C (0.0483 μg Chl h^−1^). Consistent with previous studies, this Antarctic green alga failed to grow at temperatures of 20°C or higher, confirming its psychrophilic nature (Morgan-Kiss et al., 2006; Cvetkovska et al., 2022).

**Figure 2:**
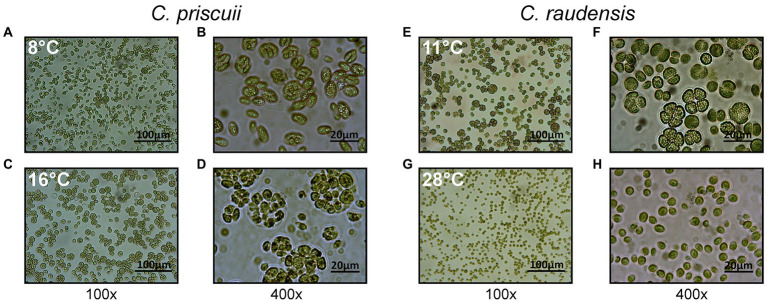
Light microscope images of both *C. priscuii* **(A–D)** and *C. raudensis* SAG 49.72 **(E–H)** cells grown under various steady-state temperatures, demonstrating the effects of growth temperature on cell morphology. The psychrophilic strain, *C. priscuii* exhibits a high ratio of palmelloids to single cells at its upper, suboptimal temperature of 16°C (C, D), compared to 8°C **(A,B)**, whereas the mesophilic *C. raudensis* SAG 49.72 strain shows this phenotype at its lower, suboptimal temperature of 11°C **(E,F)**, compared to 28°C **(G,H)**.

The growth data were complemented with an assessment of the effects of steady-state growth temperature on cell morphology, as measured by the percentage of palmelloids versus single cells present in each sample using quantitative light microscopy (Figures 1D, 2A–D).

Measurements taken shortly after cell inoculation were variable but showed little difference among the 2 different growth temperatures in which palmelloids made up approximately 25% of the population during the first 200 h of growth. *C. priscuii* grown at 8°C showed minimal changes in palmelloid content throughout the growth period with a proportion which remained fairly constant at 22–25% palmelloids even after 350–400 h (Figures 1D, 2A,B). However, we noted that the palmelloids observed at 8°C appeared to be smaller and made up of fewer cells than those observed for *C. priscuii* at 16°C (Figures 2B,C).

In contrast, the growth of *C. priscuii* at 16°C stimulated the accumulation of palmelloids relative to single cells which was most pronounced following mid-exponential growth phase which occurred at approximately 350 h (Figure 1D). Between 350 and 400 h, the palmelloid to single cell ratio was highest where palmelloids made up 43–51% of the population (Figures 1D, 2B,D). Thus, based on growth rates, it appears that the psychrophile *C. priscuii* is stressed when exposed to warm temperatures (16°C) compared to 8°C. This is correlated with a predisposition to shift cell morphology from single cells to multicellular palmelloids in *C. priscuii*.

Do mesophiles respond in a similar manner to varying growth temperature? To address this question, we examined the temperature responses of two mesophiles, *Chlamydomonas raudensis* SAG 49.72 (Figures 2E–H) and the model green alga, *Chlamydomonas reinhardtii* 1690 (Supplementary Figure 2) with respect to the response of cell morphology to growth temperature. Previous studies have established that optimal growth temperature for *C. priscuii* is near 8°C and 28°C for SAG 49.72 and *C. reinhardtii* 1690 (Morgan et al., 1998; Szyszka et al., 2007). The highest permissive growth temperature for the psychrophile is between 16° and 18°C, whereas the lowest temperature at which the mesophiles, SAG 49.72 and *C. reinhardtii* (1690), grow is between 10° and 12°C (Morgan et al., 1998, Szyszka et al., 2007). Although the mesophiles SAG 49.72 and *C. reinhardtii* 1690 also show varying cell morphology in response to growth temperature, their specific responses to temperature change is distinct compared to *C. priscuii*. Growth of SAG 49.72 and *C. reinhardtii* 1690 at their optimal temperature of 28°C resulted in cell populations composed of more than 90% single cells, whereas exposure to their lowest, permissive growth temperature of 11° to 12°C induced palmelloid formation which comprise more than 60% of the cell population (compare Figures 2F,H; compare Supplementary Figures S2E,F). Therefore, the highest permissive temperature of 16°C for the psychrophile and the lowest permissive temperature of 11° to 12°C for the two mesophiles generated the highest ratios of palmelloids to single cells. The lower growth rates indicate that these growth conditions are stressful for all three Chlamydomonas species.

### Isolation of palmelloids and single cells

**Figure 3:**
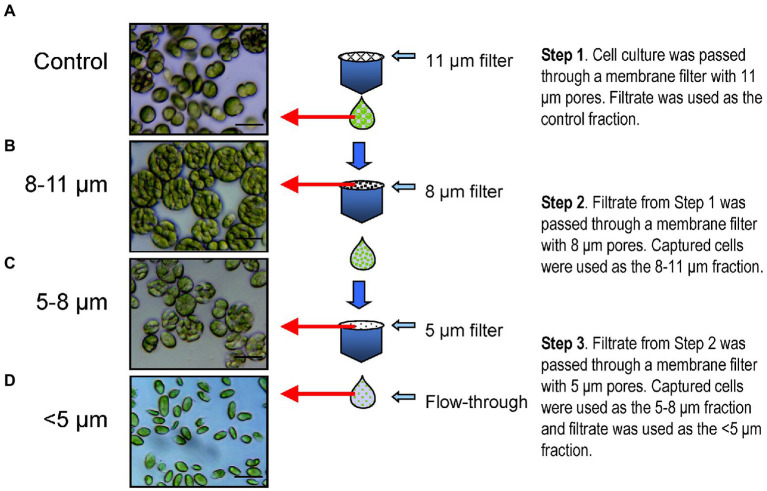
Membrane filtration procedure carried out for the separation of different sized cell units. Step 1. Dilute cell culture was passed through 11 μm hydrophilic nylon net filters by gravity flow, using a glass filter holder. Cells captured on the filter were discarded and the flow-through filtrate was utilized as the control fraction **(A)**. This control cell suspension was passed through hydrophilic polycarbonate membrane filters with 8 μm pores with the aid of gentle vacuum aspiration. Cells retained on the filters were washed with culture medium, re-suspended and centrifuged to concentrate the cells, which were used as the 8–11 μm fraction **(B)**. The flow-through filtrate was passed through hydrophilic polycarbonate membrane filters with 5 μm pores using gentle vacuum aspiration. Cells retained on the filters were washed, resuspended in culture medium and centrifuged as for the previous step, and used as the 5–8 μm fraction **(C)**. The flow-through filtrate was centrifuged and used as the single cell enriched, <5 μm fraction **(D)**. Fractions were monitored using light microscopy (**A–D**, left). Scale bars, 20 μm.

We established a technique whereby the heterogeneous mixture of control cells of *C. priscuii* grown at 8°C were separated physically based on size into isolated palmelloid and single cell fractions (Figure 3). Culture filtration using microfilters of varying pore sizes allowed for the isolation of three distinct cell population sizes: cells in the range of either 8-11 μm, 5–8 μm or those smaller than 5 μm (Figure 3). Microfilters with large, 11 μm pores removed any adherent cells as well as potential debris from the sample. The resulting filtrate was composed of a heterogeneous mixture of cells, designated as the control sample (Figure 3A). Membranes with 8 μm pores were used to isolate the largest palmelloids in the culture (Figure 3B). The filtrate from this step was then passed through membrane filters with 5 μm pores, which captured small- to medium-sized palmelloids as well as a number of large single cells (Figure 3C). The resulting filtrate contained a homogeneous suspension of single, motile cells (Figure 3D).

### Flow cytometry

**Figure 4:**
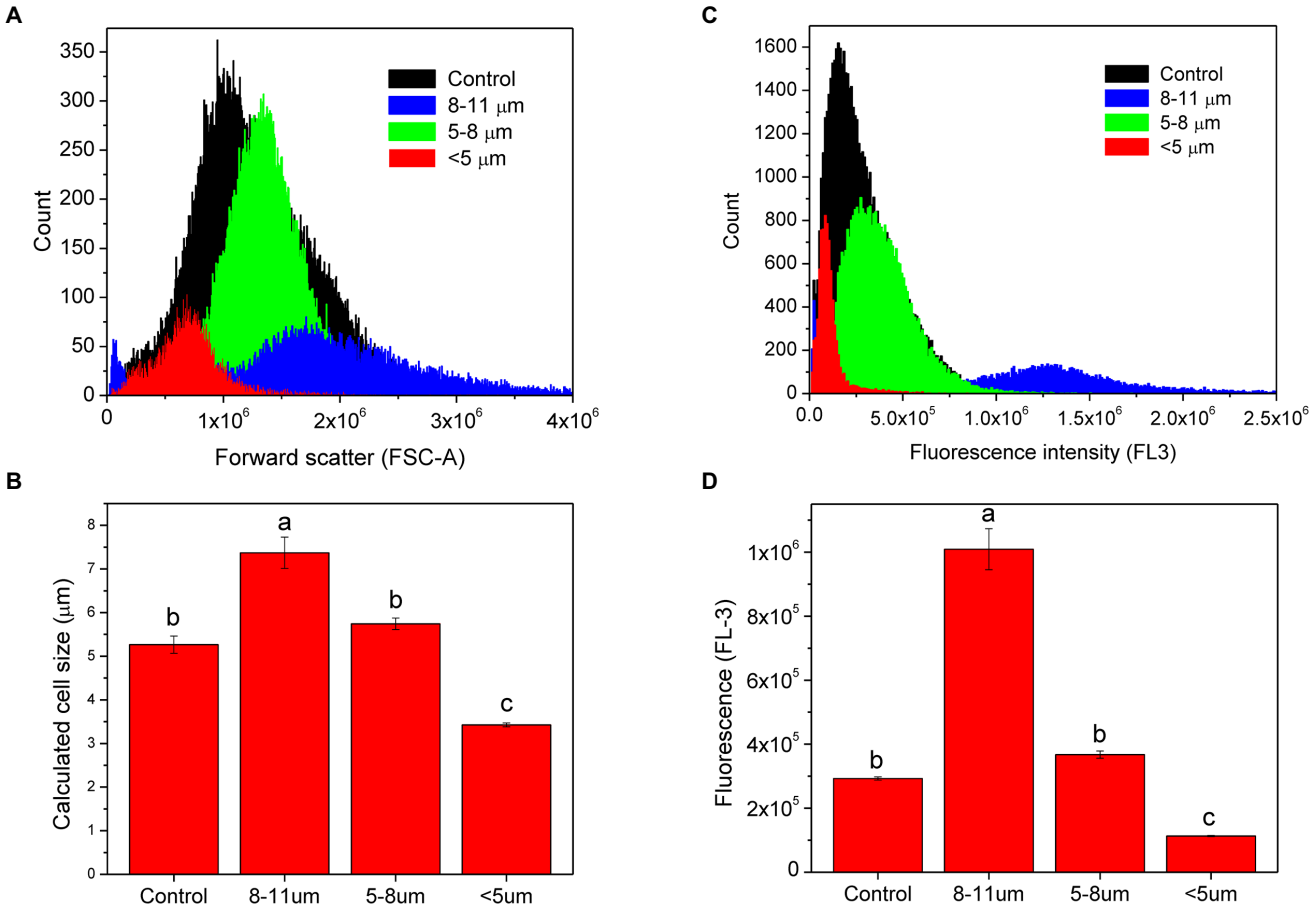
Analysis of isolated subpopulations of *C. priscuii* cells using flow cytometry. Overlay histogram of forward scatter channel (FSC-A) analysis for all cell fractions **(A)**. Estimation of cell size using mean FSC-A values and bead calibration **(B)**. Mean (± SE, *n* = 3) calculated sizes with different letters indicate significant difference, as determined by Tukey’s HSD (*p* < 0.01). Combined histograms of FL-3 (>670 nm long-pass filter), measuring red chlorophyll fluorescence **(C)** and the corresponding mean FL-3 values (± SE; Tukey’s HSD, *p* < 0.01; **D**).

Flow cytometry was used to confirm the cell size distribution and estimated cell sizes of the three different isolated fractions compared to unfractionated control cells (Figure 4). Mean forward angle light scatter (FSC) values, which are proportional to the cross-sectional area of cells, were derived from distribution curves and showed considerable differences among all 3 cell fractions (Figure 4A). Control cultures exhibited mean FSC size distribution values of 1.24 × 10^6^. Fractions consisting predominantly of large palmelloids (8–11 μm) showed mean FSC values of 1.86 × 10^6^, smaller palmelloids (5–8 μm) had mean FSC values of 1.38 × 10^6^, while the nearly homogeneous suspension of single cells resulted in a mean of 0.70 × 10^6^ (Figure 4D). Thus, a 2-fold increase in the average FSC values was observed in smaller palmelloids (5–8 μm) compared to single cells (>5 μm), while larger palmelloids (8–11 μm) showed nearly 3-fold higher mean values (Figure 4A). Therefore, the mean cross-sectional area of palmelloids was approximately 3 times that of single cells.

Estimation of the average cell size within each fraction was performed by comparing FCS values from microspheres of known sizes through the generation of a calibration curve (Figure 4B). Average diameter of cells within each fraction sizes was calculated to be 5.27 μm for control cultures, 7.37 μm for the fraction containing the largest palmelloids (8–11 μm), 5.74 for the smaller palmelloids (5–8 μm), and 3.42 μm for the predominantly single-cell fraction (<5 μm). Thus, based on the filter pore sizes used for cell separation, estimated cell sizes of each fraction were within expected ranges.

Red chlorophyll fluorescence was also measured for each separated cell fraction using an FL-3 detector (>670 nm; Figures 4C,D). Mean FL-3 values were derived from fluorescence distribution curves (Figure 4C). The largest palmelloids were characterized by the highest chlorophyll fluorescence (1.009 × 10^6^), which was 2.75 times greater than that of smaller palmelloids (0.367 × 10^6^), and 8.9 times greater than that of single cells (0.113 × 10^6^). Control cells exhibited chlorophyll fluorescence of 0.293 × 10^6^ (Figure 4D). Therefore, the variance in chlorophyll fluorescence (FL-3) among fractionated cells demonstrated greater differences, compared to their cross-sectional area (FSC).

## Photosynthetic characteristics

### 77 K fluorescence

**Figure 5:**
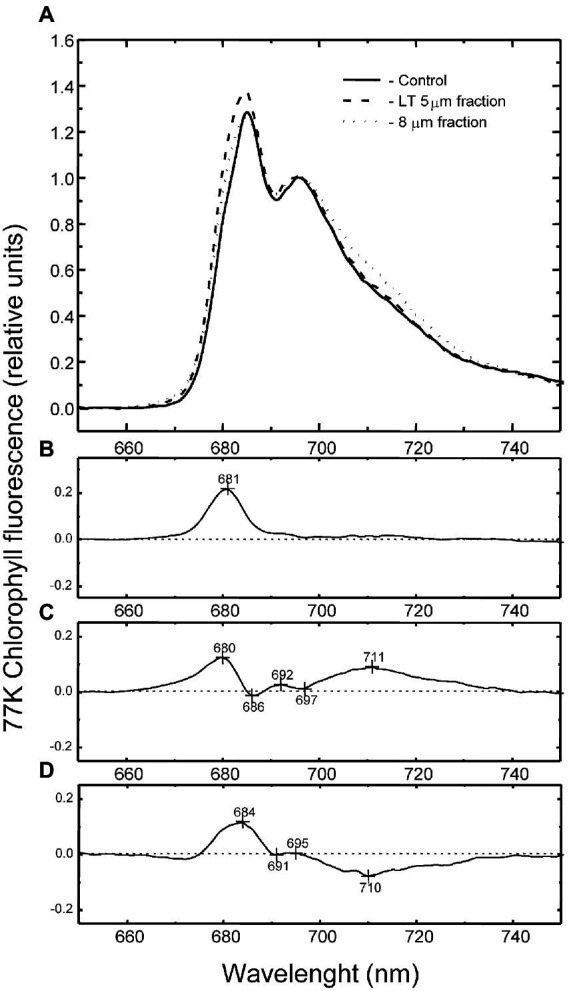
Low-temperature (77 K) chlorophyll fluorescence emission spectra of *C. priscuii* control cells (solid line), LT 5 μm cell fraction (dashed line), and 8 μm cell fraction (dotted line; **A**). Chl fluorescence was excited at 436 nm. The spectra were normalized at 695 nm. Corresponding difference spectrum of LT 5 μm cell fraction - control **(B)** 8 μm cell fraction - control **(C)** and LT 5–8 μm cell fraction **(D)**.

To assess differences in steady-state energy distribution between PSI and PSII in the thylakoids isolated from the cell fractions, we collected low-temperature (77 K) fluorescence emission spectra for control, single cells (<5 μm), and palmelloids (8–11 μm; Figure 5A; Supplementary Figure S3). Control cells exhibited the unusual 77 K fluorescence emission spectrum typical for *C. priscuii* with maxima at 682 and 696 nm associated with photosystem II but with minimal fluorescence emission in the region of 713 nm, associated with photosystem I (Morgan et al., 1998, Morgan-Kiss et al., 2002, Szyszka et al., 2007; 2015; Kalra et al., 2020). Although PSI-associated fluorescence (713 nm) was generally similar to control fractions, difference spectra indicated that single cells (<5 μm) exhibited a higher emission at 681 nm than control cells (Figures 5A,B). In contrast, palmelloids (8–11 μm) showed a higher emission at 680 nm, in addition to enhanced emission in the region of 711–713 nm (Figures 5A,C) than the control fractions. Compared to palmelloids, single cells exhibited a higher emission 680 nm, but a lower emission at 713 nm (Figure 5D; Supplementary Figure S3; Supplementary Table S1). Thus, comparative 77 K fluorescence spectroscopy of *C. priscuii* single cells versus palmelloids indicated detectable differences in energy distribution between PSII and PSI. Thus, growth morphology appears to result in measurable changes in the organization of the PSI and PSII pigment-protein complexes localized within the chloroplast thylakoid membranes of smaller single, motile cells versus the larger, multicellular palmelloids.

### Chlorophyll *a* fluorescence induction

To assess functional differences in photosynthetic characteristics, we compared room temperature fluorescence induction curves for control, single cell and palmelloid fractions measured at their growth temperature and irradiance (Table 1). Maximal PSII photochemical efficiency, measured as F_v_/F_m_, was minimally different between purified single cells (0.671 ± 0.007) and palmelloids (0.665 ± 0.020; Table 1). In addition, we quantified the following photosynthetic parameters during steady-state photosynthesis: excitation pressure, measured as 1-qL, yield of PSII (Φ_PSII_), yield of non-photochemical quenching (Φ_NPQ_), and yield of non-regulated dissipation of excess energy (Φ_NO_) and electron transport rates (ETR; Table 1). Although purified single cells and palmelloids exhibited no significant differences in maximum photochemical efficiency of PSII measured as F_v_/F_m_, both excitation pressure (1-qL) and non-photochemical quenching (Φ_NPQ_) were higher in single cells than palmelloids. However, non-regulated dissipation of excess energy (Φ_NO_) was significantly higher in palmelloids than single cells which reflects a 40% higher ratio of Φ_NO_/Φ_NPQ_ (Table 1). Thus, palmelloids preferentially dissipate excess energy through non-regulated energy quenching pathways rather than through regulated NPQ.

**Table 1:** Energy partitioning parameters calculated for each cell fraction from Chl fluorescence induction traces (Figure 6).

Samples	F_v_/F_m_	1-qL	Ф_PSII_	Ф_NPQ_	Ф_NO_	Ф_NO_/Ф_NPQ_	ETR
							
Control	0.73 ± 0.02a	0.46 ± 0.01a	0.35 ± 0.01a	0.32 ± 0.01a	0.33 ± 0.01c	1.03 ± 0.02b	14.7 0.4a
Single cells	0.67 ± 0.01b	0.47 ± 0.02a	0.3 ± 0.01b	0.35 ± 0.01a	0.36 ± 0.01b	1.02 ± 0.02b	12.6 0.4b
Palmelloids	0.67 ± 0.02b	0.39 ± 0.01b	0.34 ± 0.01a	0.27 ± 0.02b	0.38 ± 0.01a	1.41 ± 0.04a	14.2 0.4a

Values represent the mean relative yields (±SE *n* = 6) of F_v_/F_m_ - maximum photochemical efficiency, 1-qL - excitation pressure, Ф_PSII_ - efficiency of PSII, Φ_NPQ_ - non-photochemical quenching, Ф_NO_ - non-regulated energy dissipation and electron transport rates - ETR. Values followed by different letters are significantly different, as determined by ANOVA and Tukey’s test (*p* < 0.05).

**Figure 6:**
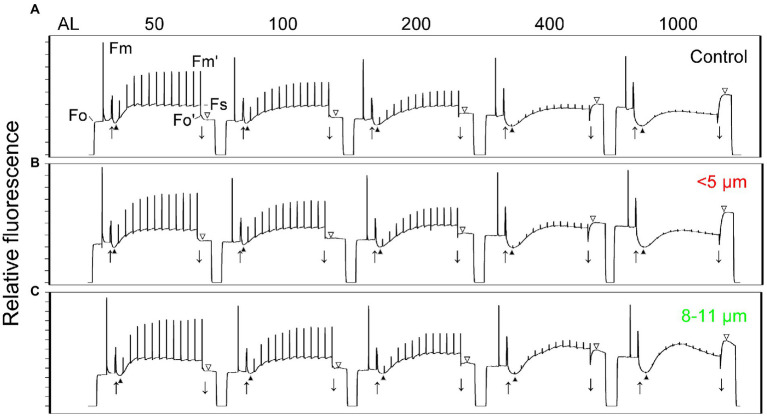
Chlorophyll fluorescence light response traces of *C. priscuii* control cells **(A)** and separated cell fractions: <5 μm **(B)** and 8–11 μm **(C)**. Cultures were measured at increasing actinic light (AL) intensities of 50, 100, 200, 400, and 1,000 μmol photons m^−2^ s^−1^. (↑) Actinic light on. (↓) Actinic light off. Fluorescence quenching during the onset of actinic light [Fo-▲], and post-illumination rise [▼-Fo’]. Cells were dark adapted for 15 min prior to collection of the fluorescence traces.

**Figure 7:**
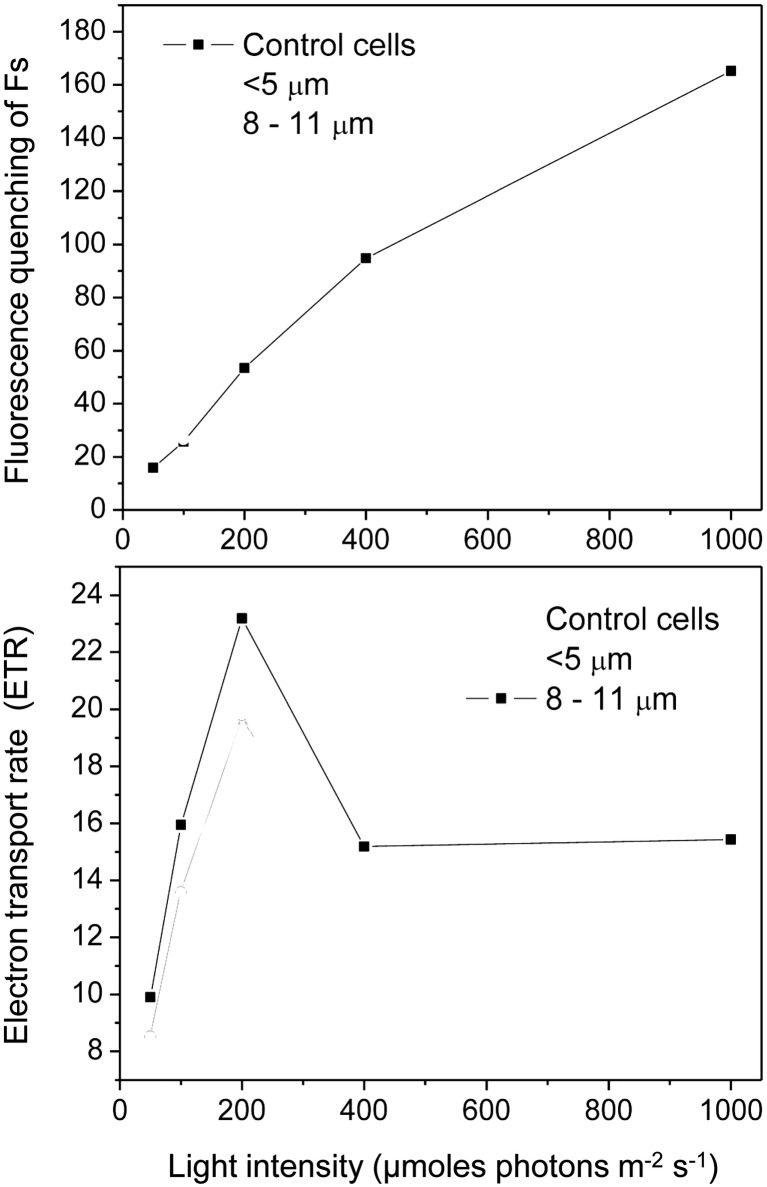
Fluorescence quenching **(A)** and electron transport rates **(B)** calculated from chlorophyll fluorescence light response traces (Figure 6) of *C. priscuii* control cells and separated cell fractions, measured at increasing actinic light (AL) intensities of 50, 100, 200, 400 and 1,000 μmol photons m^−2^ s^−1^.

Figure 6 illustrates fluorescence induction curves for cell fractions as a response to increasing actinic light intensity, ranging from 50 to 1,000 μmol photons m^−2^ s^−1^. Control cells (Figure 6A, black triangle), isolated single cells (Figure 6B, black triangle) as well as isolated palmelloids (Figure 6C, black triangle) were characterized by a fast, initial suppression of F_s_ to, or below F_o_ levels upon illumination. The extent of this initial quenching of F_s_ was quantified and plotted as a function of actinic irradiance (Figure 7A). In all cases, the extent of initial F_s_ quenching increased with increasing actinic irradiance followed by a recovery of F_s_. However, the ratio of this initial, transient quenching of F_s_ at high (1,000 μmol photons m^−2^ s^−1^) relative to low irradiance (50 μmol photons m^−2^ s^−1^) was greatest for isolated palmelloids (13.0) than for isolated single cells (7.2) which was similar to control cells (8.0). Consistent with previous results for heterogeneous populations of *C. priscuii* (Szyszka et al., 2007), increasing actinic irradiance suppressed F_m′_ similarly in all three cell fractions (Figure 6).

All cell fractions of *C. priscuii* exhibited a post-illumination rise in F_o_ (Figure 6, open triangles), the extent of which was light-dependent and comparable to the three cell fractions (Supplementary Figure S1A). However, isolated palmelloids exhibited higher Φ_NO_ quenching relative to Φ_NPQ_ (Φ_NO_/Φ_NPQ_ = 1.41) than isolated single cells (Φ_NO_/Φ_NPQ_ = 1.02; Table 1). Indeed, palmelloids exhibited lower NPQ values compared to single cells (Supplementary Figure S4). Therefore, purified palmelloids of *C. priscuii* appear to be predisposed to dissipate a greater proportion of their excess excitation energy through non-regulated pathways (NO) rather than through the typical regulated NPQ pathway compared to isolated single cells.

Although all cell fractions showed a similar increase in excitation pressure, measured as 1-qL, concomitant with a comparable decrease in PSII efficiency (Φ_PSII_) as a function of increasing irradiance (Supplementary Figures S1B), isolated single cells exhibited a greater inhibition of ETR than either isolated palmelloids and control cells at an actinic irradiance greater than 200 μmol photons m^−2^ s^−1^ (Figure 7B). At 1000 μmol photons m^−2^ s^−1^, ETR of single cells was inhibited by about 50% compared to palmelloids (Figure 7B). Thus, the palmelloid organization appears to confer greater photoprotection of the photosynthetic apparatus than observed for single cells.

### Pigment composition

**Figure 8:**
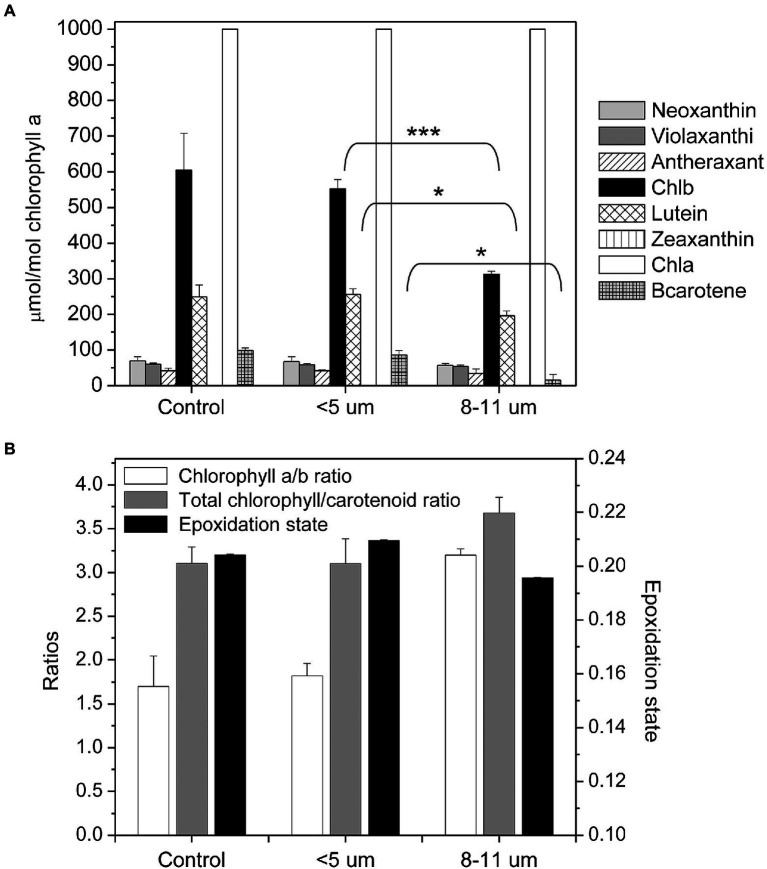
Composition of photosynthetic pigments in control and separated cell fractions of *C. priscuii* based on size **(A)**. Pigment concentrations are expressed as μmol per mole of chlorophyll a. Mean values between single cells and palmelloids were analyzed by an unpaired t-test, with the following significance levels: ^*^significant at *p* < 0.05, ^**^significant at *p* < 0.005, and ^***^significant at *p* < 0.001. No asterisk indicates no significance. Analysis of chlorophyll a/b ratios, total chlorophyll (a + b) to total carotenoid pool and epoxidation states (mean ± SE; **B**).

Pigment compositions of the isolated fractions of *C. priscuii* were quantified on a per Chl basis using HPLC (Figure 8A). Although Chla remained constant in all fractions, Chl*b* levels were 43% lower in isolated palmelloids than single cells (Figure 8A) which resulted in more than 2-fold higher Chl *a*/*b* ratio in palmelloids compared to single cells (Figure 8B). In addition, isolated palmelloids exhibited a significant 82% lower β-carotene level and a 20% lower lutein content compared to isolated single cells (Figure 8A).

**Figure 9:**
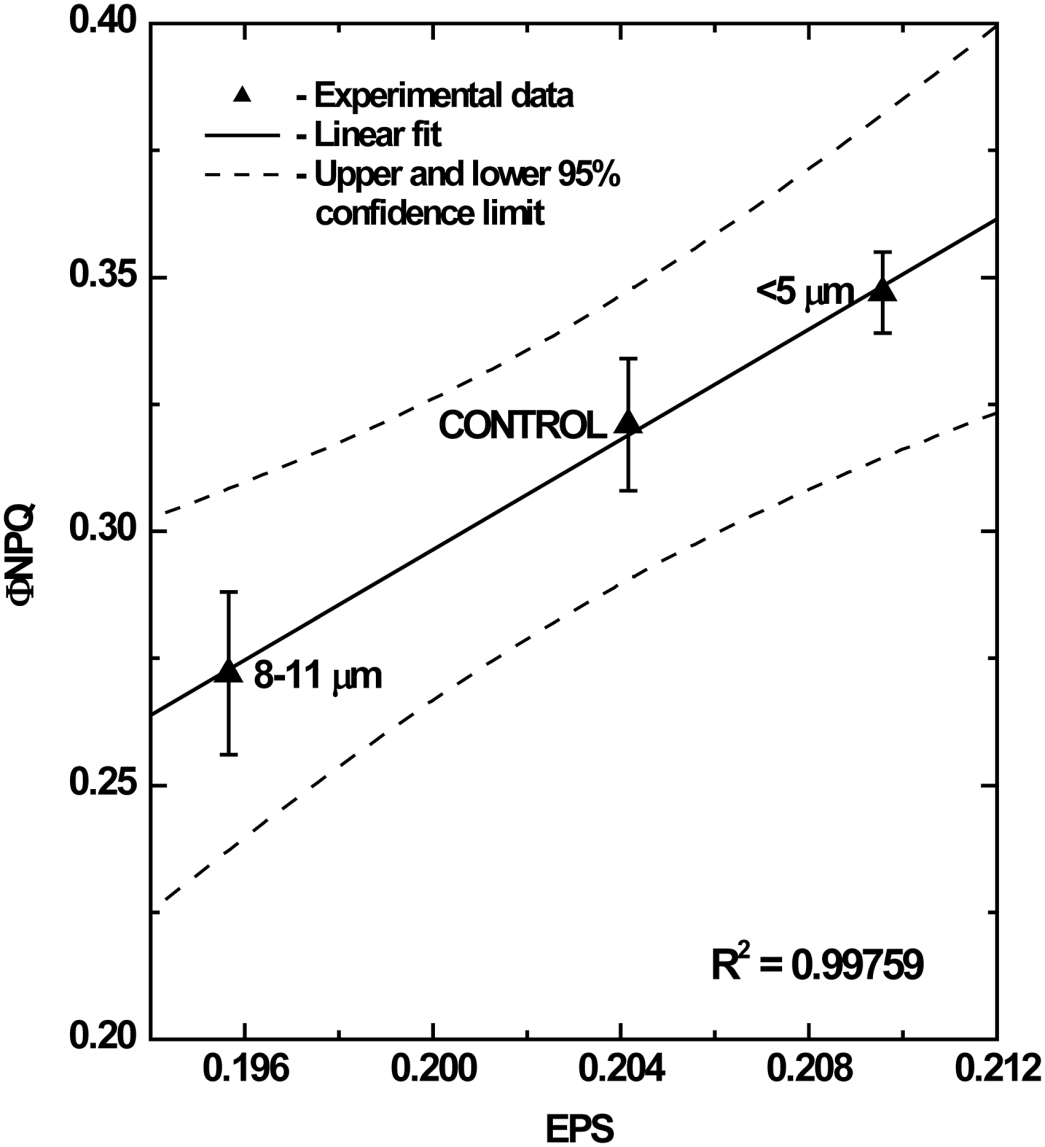
Correlation between epoxidation state (EPS) of chloroplast xanthophylls and Φ_NPQ_ in different cell fractions of *C. priscuii* grown at grown under steady-state temperatures of 8°C. All values represent means ± SE from 4 independent measurements.

Changes in the epoxidation states (EPS) of the xanthophyll cycle pigments (violaxanthin, antheraxanthin, and zeaxanthin) are known to reflect photoprotection through regulated NPQ in algae and terrestrial plants (Demmig-Adams and Adams, 1993, 1996; Demmig-Adams et al., 1999; Krol et al., 1999; Verhoeven, 2014). When we plotted Φ_NPQ_ as a function of EPS of the various isolated cell fractions we observed a very strong, positive correlation between cellular organization and Φ_NPQ_ (Figure 9). Highest Φ_NPQ_ was correlated with highest EPS which indicates that photoprotection in the single cells probably occurs primarily through the typical regulated pathway involving the xanthophyll cycle. In contrast, the lower EPS exhibited by the multicellular palmelloids is correlated with the lowest Φ_NPQ_ (Figure 9) which indicates that palmelloids do not use the xanthophyll cycle as the primary mechanism for photoprotection. These data are consistent with the higher Φ_NO_/Φ_NPQ_ ratio exhibited by palmelloids than single cells (Table 1). This is consistent with the thesis that photoprotection in multicellular palmelloids is distinguishable from that observed in single cells of the same algal species.

### SDS-PAGE and immunoblotting

**Figure 10:**
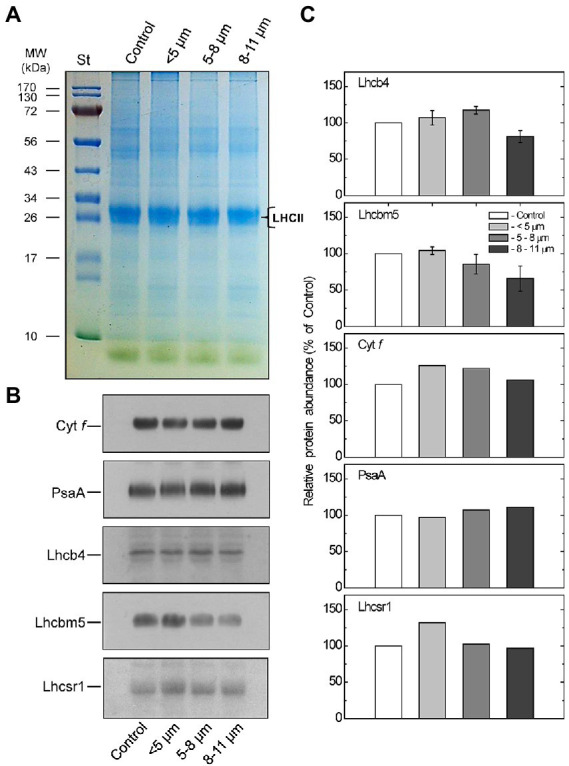
Coomassie stained SDS-PAGE analysis of thylakoid membrane proteins from control and different-sized cell fractions of *C. priscuii* **(A)**. Lanes of SDS-PAGE were loaded on an equal Chl basis. Values on the left indicate apparent molecular mass (kDa). B – Representative Western blots **(B)** and densitometric analysis **(C)** of polypeptides probed with antibodies raised against Cyt f, Lhcbm5, Lhcb4, and PsaA polypeptides in thylakoid membranes isolated from different-sized cell fractions of *C. priscuii*. Mean values ± SE were calculated from 3 independent experiments. The presented data were normalized to the relative abundance of the respective polypeptides in the control whole cell fraction.

Pigments of the photosynthetic apparatus identified by HPLC (Figure 8) are bound to proteins that constitute the pigment-protein complexes associated with PSI and PSII including their light-harvesting complexes (LHCI and LHCII). Thylakoid membrane proteins from the isolated cell fractions were analyzed by SDS-PAGE (Figure 10A) combined with immunoblotting to assess the content of the specific LHCII polypeptides, LHCbm5 and LHCb4, photosystem I reaction centre polypeptide, PsaA and Cytf of the Cytb_6_/f complex (Figure 10B). Coomassie-stained gels of thylakoid polypeptides from each fraction showed little difference between their protein compositions although purified palmelloids (8–11 μm) appeared to contain lower levels of LHCII proteins (Figure 10A) which was confirmed by quantification of immunoblotting with antibodies specific for PSII light-harvesting polypeptides, Lhcbm5, Lhcb4 and Lhcrs1 (Figures 10B,C). The decreased abundance of Lhcbm5 and Lhcb4 polypeptides in isolated palmelloids compared to isolated single cells is consistent with increased Chl a/b ratios (Figure 8B), and decreased chlorophyll fluorescence emission at 680 nm, compared to purified single cells (Figure 5D; Supplementary Table S1). It appears also that Lhcrs1 levels are highest in single cells (Figures 10B,C), which correlates well with the observed highest Φ_NPQ_ in this cell fraction (Table 1). We have also tested our samples with anti-Lhcrs3, but unfortunately it did not cross-react with any protein band in any of the cell fractions of *C. priscuii.* In contrast, the relative levels of the PsaA and Cyt in palmelloids versus single cells remained fairly constant (Figures 10B,C).

## Discussion

Previous studies have shown that palmelloid formation in *Chlamydomonas* can be induced by altering culture media, addition of compounds which inhibit growth, malfunction in flagella and cell walls, and in the presence of grazers (Iwasa and Murakami, 1968; Nakamura et al., 1975; Mikheeva and Kruchkova, 1980; Olsen et al., 1983; Devine et al., 1993; Franqueira et al., 1999; Lurling and Beekman, 2006; Neelam and Subramanyam, 2013). However, to our knowledge, induction of the palmelloid state in *Chlamydomonas* has not been previously reported as a response to growth temperature.

In both the Antarctic psychrophile, *Chlamydomonas priscuii* and the mesophiles *Chlamydomonas raudensis* SAG 49.72 and the model *Chlamydomonas reinhardtii* 1690, temperature had a profound effect on cell morphology. Exposure to suboptimal growth temperatures of 11–12°C for the mesophilic species *Chlamydomonas raudensis* SAG 49.72 and *Chlamydomonas reinhardtii* 1,690 not only reduced growth rates (Szyszka et al., 2007) but also induced a high ratio of multicellular palmelloids to single cells (Figures 1D, 2; Supplementary Figure S2). Analogously, exposure of psychrophilic *C. priscuii* to high temperature (16°C) inhibited its growth rate and induced the accumulation of palmelloids versus single cells (Figure 1D). Thus, we conclude that palmelloid formation in Chlamydomonas species appears to be induced by either low temperature in mesophilic species (*C. raudensis* SAG 49.72 and *C. reinhardtii* 1,690) or high-temperature stress in psychrophilic species (*C. priscuii*). Thus, we suggest that temperature stress must generate an important developmental signal that governs multicellular palmelloid formation in Chlamydomonas species. However, major changes in phenotype in response to temperature stress are not restricted to algae. High-temperature stress has been reported to induce a change in phenotype in psychrophilic fungi called snow molds from a vegetative hyphal state to a dormant, sclerotial form which enables survival of the fungus at a high, non-permissive growth temperature (Newsted and Huner, 1987, 1988).

Physical separation of the single cells and palmelloids of *C. priscuii* allowed us to assess independently their photosynthetic characteristics. Analysis of room temperature Chl *a* fluorescence of single cells versus palmelloids of *C. priscuii* demonstrated that under optimal growth conditions, maximum photochemical efficiency (F_v_/F_m_) differed minimally (Table 1). However, the rapid, fluorescence quenching observed as a function of increasing irradiance was greater in single cells compared to palmelloids (Figure 7A). This indicates that during the initial illumination immediately after dark adaptation, the plastoquinone pool of single cells remains more oxidized than that of palmelloids (Figure 7A). However, the post-illumination rise in fluorescence yield after steady-state photosynthesis that had been achieved was greater in single cells than palmelloids. We interpret this to indicate that the plastoquinone pool of single cells remained more reduced during steady-state photosynthesis compared to palmelloids (Figure 7A). These results are consistent with the greater inhibition of steady-state electron transport rates at high light for single cells than for palmelloids of *C. priscuii* (Figure 7B).

Although single cells of *C. priscuii* exhibited 29% higher Φ_NPQ_ (Table 1) and NPQ (Supplementary Figure S4) values compared to palmelloids under the same conditions (Table 1; Supplementary Figure S4), multicellular palmelloids structures concomitantly exhibited higher levels of non-regulated dissipation (Φ_NO_) resulting in a 1.4-fold higher ratio of Φ_NO_/Φ_NPQ_ (Table 1). Therefore, single cells of *C. priscuii* appear to favor partitioning of excess excitation energy through typical down-regulatory processes (NPQ) localized to the light-harvesting complexes (Horton et al., 1996, 1999; Pascal et al., 2005), while palmelloids favor partitioning of excess energy through other, constitutive energy dissipative processes (Φ_NO_). This is supported by the data illustrated in Figure 9. Although the mechanism of non-regulated, constitutive quenching (Φ_NO_) remains equivocal, it has been suggested that, in addition to antenna quenching, energy quenching may proceed *via* PSII reaction centers (Weis and Berry, 1987; Bukhov et al., 2001; Ivanov et al., 2008b).

Pigment analysis revealed that compared to single cells, palmelloids of *C. priscuii* exhibit significantly reduced levels of Chl *b*, resulting in higher Chl *a*/*b* ratios (Figures 8A,B). In addition, palmelloids exhibit lower total carotenoid pools, with a substantial reduction of β-carotene and a lesser decline of lutein (Figure 8B). Carotenoids augment light harvesting by transferring energy to chlorophylls or provide photoprotection by non-photochemical quenching (NPQ) of excess excitation energy as thermal dissipation. Another important function of carotenoids is their involvement in folding and stability of light-harvesting complexes (Plumley and Schmidt, 1987; Giuffra et al., 1996; Bassi et al., 1999). In *Arabidopsis*, the absence of xanthophylls or lutein results in alterations of photosystem antenna sizes and reduced stability of trimeric LHCII (Lokstein et al., 2002). Reduction of LHCs has also been observed in lutein-deficient green algae, *Scenedesmus* and *Chlamydomonas* (Chunaev et al., 1991; Heinze et al., 1997). As a result, lutein-deficient mutants of *Arabidopsis* (*lut1* and *lut2*) and *Chlamydomonas* (*lorl*) demonstrate low levels of NPQ and a delay in the induction of NPQ (Niyogi et al., 1997; Pogson et al., 1998; Popova et al., 2019). Thus, decreased lutein and overall carotenoid pool size in palmelloids of *C. priscuii* may contribute to the lower abundance of LHCII proteins (Figure 10) and lower levels of NPQ (Table 1; Supplementary Figure S4). This is consistent with the lower abundance of Lhcb4 and Lhcbm5 (Figure 10), greater Chl a/b ratios (Figure 8B), and decreased F682/F712 ratios (Figure 5D; Supplementary Table S1) in palmelloids compared to single cells. Φ_NPQ_ is dependent on xanthophyll cycle activity and light-harvesting proteins (Demmig-Adams and Adams, 1993, 1996; Demmig-Adams et al., 1999; Pascal et al., 2005; Ruban et al., 2007; Murchie et al., 2009). Therefore, a reduction of both carotenoid pools and LHCII proteins may result in a reduction of Φ_NPQ_ through antenna quenching in palmelloids of *C. priscuii*. We suggest that the lower level of NPQ *via* antenna quenching in palmelloids is compensated by the observed increased level of non-regulated dissipation of excess energy (NO; Table 1; Figure 9) *via* reaction center quenching (Zulfigarov et al., 2007; Ivanov et al., 2008a,b). Previous results for the Chl *b*-deficient c*hlorina F2* barley mutant support this thesis. The c*hlorina F2* mutant exhibits an extensively reduced light-harvesting antennae size which is associated with a 38% lower Φ_NPQ_ coupled with a 34% increase in constitutive, non-regulated energy dissipation (Φ_NO_), presumed to occur *via* reaction center quenching (Ivanov et al., 2008a). In addition, the lower abundance of Lhcrs1, one of the regulators of NPQ in Chlamydomonas species (Dinc et al., 2016) in palmelloids (Figures 10B,C) supports our contention that photoprotection in palmelloids is primarily a function of non-regulated NPQ rather then regulated NPQ. Unfortunately, our attempt to identify and quantify the key NPQ regulator in Chlamydomonas, i.e., Lhcrs3 (Peers et al., 2009) using anti-Lhcrs3 from Agrisera was not successful. The antibody did not cross-react with *C. priscuii* protein samples.

In the present study, large palmelloids of *C. priscuii* maintained the high levels of non-regulated energy dissipation (NO) and lower levels of non-photochemical quenching (NPQ) during steady-state growth and exposure to high irradiance (Figure 6; Table 1). Previous studies (Szyszka et al., 2007) have reported that compared to growth at low temperatures, high levels of Φ_NO_ and reduced Φ_NPQ_ were also exhibited by *C. priscuii* cultures grown at 16°C, where large palmelloids make up nearly half of the population (Figures 1D, 2D). Thus, a novel observation is that the regulation of partitioning of excess light energy appears to be closely related to the organization of *C. priscuii* cells.

The formation of palmelloids as a function of growth temperature is not restricted to the psychrophile, *C. priscuii,* which is obligately adapted to low temperature (4°C; Morgan-Kiss et al., 2006). The mesophilic Chlamydomonas species *C. raudensis* (Figure 2F) as well as *C. reinhardtii* (Supplementary Figures S2E,F) adapted to warm temperatures (28°C) also form palmelloids in response to changes in growth temperature. Despite the obvious differences in optimal growth temperatures, we suggest that the formation of palmelloids in the mesophiles and the psychrophile represent a response to temperature stress. The exposure of the psychrophile, *C. priscuii*, to high growth temperatures (16°C) that are close to non-permissive conditions (18–20°C) are stressful for the psychrophile. In response, *C. priscuii*, forms palmelloids to photoprotect its photosynthetic apparatus through a reduction in light absorption due to less abundant PSII light-harvesting complexes coupled with enhanced energy dissipation of excess absorbed light *via* NO quenching mechanisms. Thus, the photosynthetic apparatus within a palmelloid is subtly different from that within a single cell. We conclude that Chlamydomonas species respond to temperature stress by inducing a developmental change from motile, single cells to immotile, large palmelloids, in part, to protect the photosynthetic apparatus from photoinhibition.

It may be argued that palmelloid formation can cause an intra-palmelloid shading effect of chloroplasts which would reduce light absorption and protect the chloroplast from excess light energy. Typically, a shading effect should decrease Chla/b ratios and increase light-harvesting polypeptide content (Anderson et al., 1995). However, we observed the opposite effect in isolated palmelloids relative to single cells of *C. priscuii*: a doubling in the Chla/b ratio (Figure 8), a decrease in carotenoid content coupled with a decrease in LHCII polypeptides content (Figure 10). Functionally, these biochemical differences are correlated with a 40% enhancement in energy dissipation in palmelloids relative to single cells specifically through non-regulated energy quenching mechanisms (Φ_NO_; Table 1) and a reorganization of PSII and PSI as detected by difference spectra of 77 K fluorescence emission (Figure 5). Thus, the photosynthetic apparatus of cells trapped within an immotile palmelloid of *C. priscuii* is subtly different from that within a single, motile cell. We conclude that *C. priscuii* responds to temperature stress not only by inducing a phenotypic change from motile, single cells to immotile, colonial palmelloids but, in the process, also modulates the biochemical composition, organization, and function of the photosynthetic apparatus to protect it from the absorption of excess light energy. Thus, we conclude that palmelloid formation in the cold-adapted, Antarctic psychrophile, *Chlamydomonas priscuii*, is photoprotective. The mechanism that governs this complex response to temperature change at the cellular and molecular levels remains to be elucidated.

## Data availability statement

The raw data supporting the conclusions of this article will be made available by the authors, without undue reservation.

## Author contributions

All authors listed have made a substantial, direct, and intellectual contribution to the work and approved it for publication.

## Funding

NH is grateful for the research support from an NSERC Discovery Grant and the past support through the Canada Foundation for Innovation and the Canada Research Chairs program. This work was also funded by NSERC Discovery Grant 4458-2016 awarded to CT.

## Conflict of interest

The authors declare that the research was conducted in the absence of any commercial or financial relationships that could be construed as a potential conflict of interest.

## Publisher’s note

All claims expressed in this article are solely those of the authors and do not necessarily represent those of their affiliated organizations, or those of the publisher, the editors and the reviewers. Any product that may be evaluated in this article, or claim that may be made by its manufacturer, is not guaranteed or endorsed by the publisher.

## Footnotes

^1^ http://rsb.info.nih.gov.ij

## Supplementary material

Supplementary Figure S4 Light response of non-photochemical quenching (NPQ) of *C. priscuii* control cells (black) and separated cell fractions: <5 μm (red), 5–8 μm (green), and 8–11 μm (blue). Cultures were measured at increasing actinic light (AL) intensities of 50, 100, 200, 400, 1,000, and 2000 μmol photons m^−2^ s^−1^.
