# Supplementary figures and images for: Palmelloid formation in the Antarctic psychrophile, Chlamydomonas priscuii, is photoprotective
Source: Front Plant Sci. 2022 Aug 31;13:911035. doi: 10.3389/fpls.2022.911035 (PMC9470844; doi:10.3389/fpls.2022.911035)

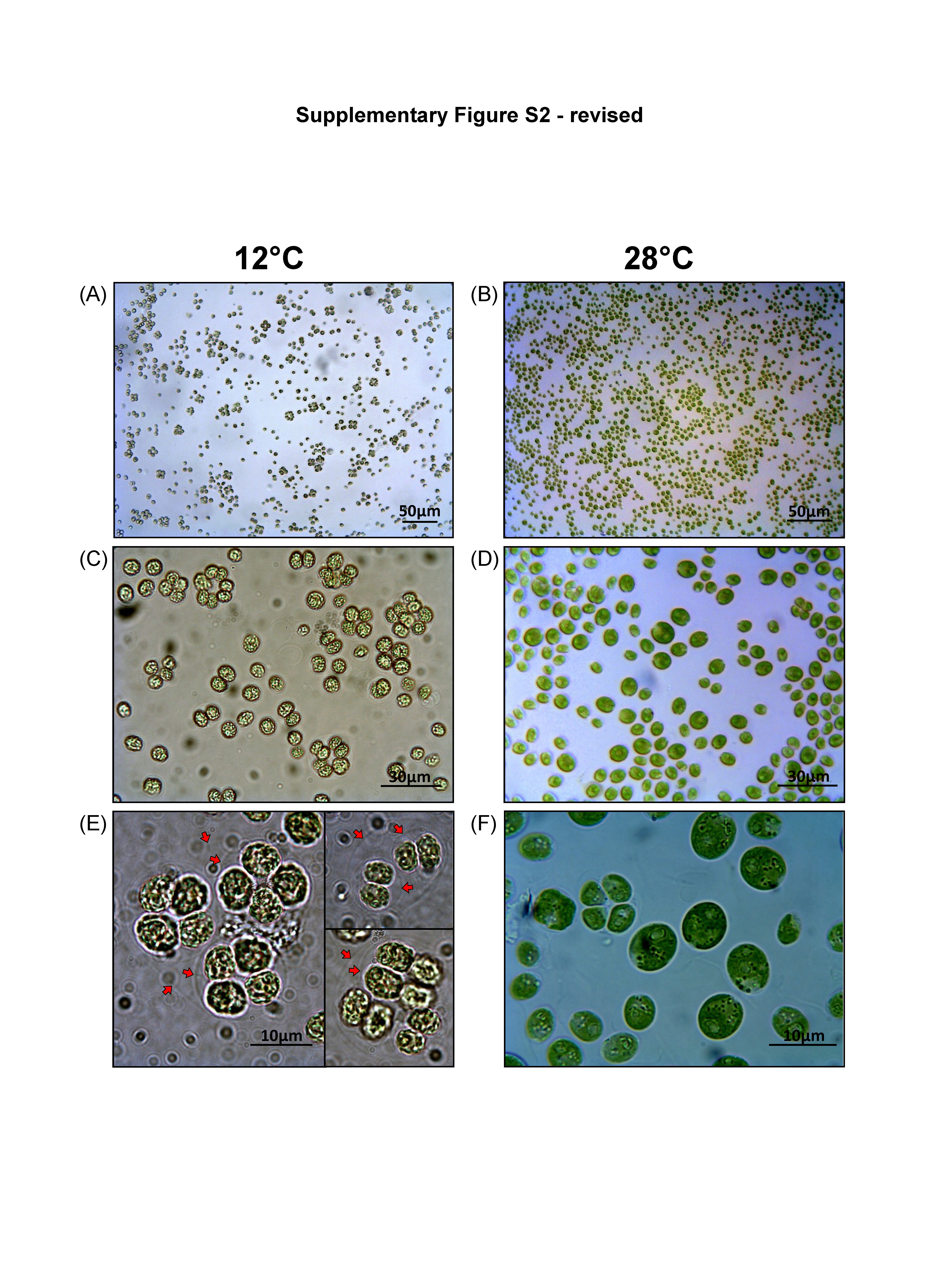

Supplement: Supplementary file 2 [file Image_2.TIFF]
