# Supplementary material for: Palmelloid formation in the Antarctic psychrophile, Chlamydomonas priscuii, is photoprotective
Source: Front Plant Sci. 2022 Aug 31;13:911035. doi: 10.3389/fpls.2022.911035 (PMC9470844; doi:10.3389/fpls.2022.911035)
Supplement: Supplementary file 5 [file Table_1.DOCX]

**Table S1**. Gaussian fitting parameters for the subband decompositions of 77K chlorophyll fluorescence spectra of whole culture (control) and fractionated cells of *C. priscuii.* The percentage areas of the spectral forms have been calculated from the total area given by the sum of all bands reported. The FWHM of each band is the sum of the left and right HWHM values. FWHM - full width at half maximum; HWHM - half width at half maximum.

| Parameters | Control | LT5 µm | 5 µm | 8 µm |
| --- | --- | --- | --- | --- |
|  |  |  |  |  |
| 1 λ max | 681. 6 | 681.6 | 681.1 | 681.5 |
| FWHM | 7.3 | 7.3 | 6.5 | 6.7 |
| Area % | 14.97 | 19.16 | 10.79 | 12.69 |
|  |  |  |  |  |
| 2 λ max | 685.7 | 685.9 | 685.5 | 685.8 |
| FWHM | 4.4 | 4.1 | 4.4 | 4.1 |
| Area % | 5.76 | 3.75 | 5.32 | 3.39 |
|  |  |  |  |  |
| 3 λ max | 694.2 | 694.3 | 694.1 | 694.2 |
| FWHM | 14.5 | 15.0 | 16.4 | 18.4 |
| Area % | 34.09 | 35.60 | 38.78 | 43.93 |
|  |  |  |  |  |
| 4 λ max | 709.9 | 711.1 | 711.8 | 713.5 |
| FWHM | 20.2 | 18.0 | 18.3 | 17.4 |
| Area % | 20.17 | 15.23 | 16.76 | 13.34 |
|  |  |  |  |  |
| 5 λ max | 730.0 | 724.5 | 724.6 | 724.6 |
| FWHM | 50.4 | 51.8 | 53.1 | 51.5 |
| Area % | 24.20 | 26.23 | 28.32 | 26.62 |
|  |  |  |  |  |
| Chi^2^ | 0.00003 | 0.00004 | 0.00005 | 0.00005 |
