# Supplementary material for: Palmelloid formation in the Antarctic psychrophile, Chlamydomonas priscuii, is photoprotective
Source: Front Plant Sci. 2022 Aug 31;13:911035. doi: 10.3389/fpls.2022.911035 (PMC9470844; doi:10.3389/fpls.2022.911035)
Supplement: Supplementary file 6 [file Table_2.DOCX]

**SUPPLEMENTARY FIGURES**

**Supplementary Figure S1.** Parameters calculated from chlorophyll fluorescence light response traces of *C. priscuii* control cells and separated cell fractions, measured at increasing actinic light (AL) intensities of 50, 100, 200, 400 and 1000 µmol photons m^-2^ s^-1^. Fluorescence quenching during onset of actinic light [Fo-▲] (A), post-illumination rise [▼-Fo'] (B), 1-qL = excitation pressure (C) and ФPSII = efficiency of PSII (D).

**Supplementary Figure S2.** Light microscope images of *Chlamydomonas reinhardtii* (1690) grown at 12°C and 28°C, at 10x (A, B), 40x (C, D) and 400x (E, F) magnification. The ratio of palmelloids to single cells was greater during growth at lower temperatures. At high magnification, multiple membranes are visible (red arrows) surrounding cells grown at 12°C.

**Supplementary Figure S3.**

Low temperature (77K) chlorophyll fluorescence emission spectra and decomposition in Gaussian subbands of *C. priscuii* control cells and separated cell fractions based on size. Cultures were grown at 5°C and 100 µmol photons m^-2^ s^-1^. Cell fractions separated from exponentially growing cultures were incubated in the dark for 5 min at 5°C before being quickly frozen in liquid nitrogen. Chlorophyll fluorescence was excited at 436 nm. Experimental curves represent averages of three scans in three independent measurements.
